# Supplementary material for: The turning and barrier course reveals gait parameters for detecting freezing of gait and measuring the efficacy of deep brain stimulation
Source: PLoS One. 2020 Apr 29;15(4):e0231984. doi: 10.1371/journal.pone.0231984 (PMC7190141; doi:10.1371/journal.pone.0231984)
Supplement: Attachment — Submitted filename: Response to Reviewers.docx [file pone.0231984.s001.docx]

**Response to Reviewers: The turning and barrier course reveals gait parameters for detecting freezing of gait and measuring the efficacy of deep brain stimulation**

**Manuscript No.: PONE-D-19-35218**

We greatly appreciate these reviews, we really think they have improved the quality of the manuscript and we appreciated the reviewers’ time and energy into their reviews. Here we address each reviewer’s suggestions and questions, and we outline where the changes have been made in the revised manuscript.

**Journal Requirements:**

*(1) Please ensure that your manuscript meets PLOS ONE's style requirements, including those for file naming. The PLOS ONE style templates can be found at* [*http://www.plosone.org/attachments/PLOSOne_formatting_sample_main_body.pdf*](http://www.plosone.org/attachments/PLOSOne_formatting_sample_main_body.pdf) *and* [*http://www.plosone.org/attachments/PLOSOne_formatting_sample_title_authors_affiliations.pdf*](http://www.plosone.org/attachments/PLOSOne_formatting_sample_title_authors_affiliations.pdf)

Response: We have ensured that the manuscript meets PLOS ONE’s style requirements, including those for file naming.

*(2) Thank you for stating the following in the Financial Disclosure section:*

*"This study was supported in part by the Michael J. Fox Foundation (9605, HBS), the National Institute of Neurological Disorders and Stroke (NINDS Grant 5 R21 NS096398-02; HBS, CA, JSN), the Robert and Ruth Halperin Foundation (HBS), the John A. Blume Foundation (HBS), the Helen M. Cahill Award for Research in Parkinson's Disease (HBS), the Stanford Bio-X Bowes Graduate Fellowship (JO), and the National Institutes of Health Big Data to Knowledge (BD2K) Center of Excellence Grant U54EB020405 (SD, JO). Medtronic Inc. provided the devices used in this study but no additional financial support. The funders had no role in study design, data collection and analysis, decision to publish, or preparation of the manuscript.

Michael J Fox:* [*https://www.michaeljfox.org/*](https://www.michaeljfox.org/) *NINDS:* [*https://www.ninds.nih.gov/*](https://www.ninds.nih.gov/) *Stanford Bio-X:* [*https://biox.stanford.edu/*](https://biox.stanford.edu/) *NIH BD2K:* [*https://commonfund.nih.gov/bd2k*](https://commonfund.nih.gov/bd2k)*"*

*We note that one or more of the authors are employed by a commercial company: Cala Health**. Please provide an amended Funding Statement declaring this commercial affiliation, as well as a statement regarding the Role of Funders in your study. If the funding organization did not play a role in the study design, data collection and analysis, decision to publish, or preparation of the manuscript and only provided financial support in the form of authors' salaries and/or research materials, please review your statements relating to the author contributions, and ensure you have specifically and accurately indicated the role(s) that these authors had in your study. You can update author roles in the Author Contributions section of the online submission form.*

*Please also include the following statement within your amended Funding Statement.*

*“The funder provided support in the form of salaries for authors [insert relevant initials], but did not have any additional role in the study design, data collection and analysis, decision to publish, or preparation of the manuscript. The specific roles of these authors are articulated in the ‘author contributions’ section.”*

*If your commercial affiliation did play a role in your study, please state and explain this role within your updated Funding Statement.*

Response: At the time that the data was collected and study performed, all the authors of the study were employees and funded by Stanford University and the project was funded by the funders listed in the study. Subsequent to this, one of the authors (JSN) became employed by Cala Health. The funders did not have any role in the study design, data collection and analysis, decision to publish, or preparation of the manuscript.

*(3) Please also provide an updated Competing Interests Statement declaring this commercial affiliation along with any other relevant declarations relating to employment, consultancy, patents, products in development, or marketed products, etc.  
Within your Competing Interests Statement, please confirm that this commercial affiliation does not alter your adherence to all PLOS ONE policies on sharing data and materials by including the following statement: "This does not alter our adherence to PLOS ONE policies on sharing data and materials.” (as detailed online in our guide for authors* [*http://journals.plos.org/plosone/s/competing-interests*](http://journals.plos.org/plosone/s/competing-interests)*) . If this adherence statement is not accurate and  there are restrictions on sharing of data and/or materials, please state these. Please note that we cannot proceed with consideration of your article until this information has been declared.*

*Please include both an updated Funding Statement and Competing Interests Statement in your cover letter. We will change the online submission form on your behalf.*

Response: We have updated the declaration of the commercial affiliation in the Competing Interest statement, “Scott Delp is a scientific advisor and board member of Cala Health, Circuit Therapeutics, and Zebra Medical Technologies, and receives compensation for this service,” and noted that this does not alter our adherence to PLOS ONE policies on sharing data and materials, see response #4 below.

*(4) Thank you for stating the following in the Competing Interests section:* *"I have read the journal's policy and the authors of this manuscript have the following competing interests: Dr. Helen Bronte-Stewart is a member of a clinical advisory board for Medtronic Inc."*

*Please confirm that this does not alter your adherence to all PLOS ONE policies on sharing data and materials, by including the following statement: "**This does not alter our adherence to  PLOS ONE policies on sharing data and materials.” (as detailed online in our guide for authors* [*http://journals.plos.org/plosone/s/competing-interests*](http://journals.plos.org/plosone/s/competing-interests)*).  If there are restrictions on sharing of data and/or materials, please state these. Please note that we cannot proceed with consideration of your article until this information has been declared.*

*Please include your updated Competing Interests statement in your cover letter; we will change the online submission form on your behalf.*

*Please know it is PLOS ONE policy for corresponding authors to declare, on behalf of all authors, all potential competing interests for the purposes of transparency. PLOS defines a competing interest as anything that interferes with, or could reasonably be perceived as interfering with, the full and objective presentation, peer review, editorial decision-making, or publication of research or non-research articles submitted to one of the journals. Competing interests can be financial or non-financial, professional, or personal. Competing interests can arise in relationship to an organization or another person. Please follow this link to our website for more details on competing interests**:* [*http://journals.plos.org/plosone/s/competing-interests*](http://journals.plos.org/plosone/s/competing-interests)

Response: I have read the journal's policy and the authors of this manuscript have the following competing interests: Dr. Helen Bronte-Stewart is a member of a clinical advisory board for Medtronic Inc. and Scott Delp is a scientific advisor and board member of Cala Health, Circuit Therapeutics, and Zebra Medical Technologies, and receives compensation for this service. Dr. Helen Bronte-Stewart and Johanna O’Day have submitted a provisional patent as co-inventors of systems and methods for deep brain stimulation kinematic controllers (patent #S19-551). This does not alter our adherence to PLOS ONE policies on sharing data and materials.

*(5) We note that you have stated that you will provide repository information for your data at acceptance. Should your manuscript be accepted for publication, we will hold it until you provide the relevant accession numbers or DOIs necessary to access your data. If you wish to make changes to your Data Availability statement, please describe these changes in your cover letter and we will update your* *Data Availability statement to reflect the information you provide.*

Response: All data will be available at acceptance via the SIMTK website (<https://simtk.org/projects/pdwalking>) provided in Data Availability statement.

**Reviewers’ comments:**

Reviewer's Responses to Questions

**Comments to the Author**

1. Is the manuscript technically sound, and do the data support the conclusions?

The manuscript must describe a technically sound piece of scientific research with data that supports the conclusions. Experiments must have been conducted rigorously, with appropriate controls, replication, and sample sizes. The conclusions must be drawn appropriately based on the data presented.

Reviewer #1: Yes

Reviewer #2: Partly

2. Has the statistical analysis been performed appropriately and rigorously?

Reviewer #1: Yes

Reviewer #2: Yes

3. Have the authors made all data underlying the findings in their manuscript fully available?

The [PLOS Data policy](http://www.plosone.org/static/policies.action#sharing) requires authors to make all data underlying the findings described in their manuscript fully available without restriction, with rare exception (please refer to the Data Availability Statement in the manuscript PDF file). The data should be provided as part of the manuscript or its supporting information, or deposited to a public repository. For example, in addition to summary statistics, the data points behind means, medians and variance measures should be available. If there are restrictions on publicly sharing data—e.g. participant privacy or use of data from a third party—those must be specified.

Reviewer #1: Yes

Reviewer #2: No

4. Is the manuscript presented in an intelligible fashion and written in standard English?

PLOS ONE does not copyedit accepted manuscripts, so the language in submitted articles must be clear, correct, and unambiguous. Any typographical or grammatical errors should be corrected at revision, so please note any specific errors here.

Reviewer #1: Yes

Reviewer #2: Yes

**Review Comments to the Author**

**Reviewer #1:**
*(1) This represents an interesting piece of scientific work, clearly and accurately written, presenting new tasks and protocol to induce FoG events in a clinic-like setup. The authors argue that “TBC is easily assembled and mimics real-life environments that elicit FOG”. However, it requires some room and specific equipment to be applied. The continuous turning task, in comparison, has shown to be very sensitive in detecting freezing events while not requiring either room or special equipment. As the current results have clear potential applicability in clinical settings, it would be desirable to make a comparison between the evaluation tasks, discussing potential benefits of using TBC rather than the turning task. I understand that such an argumentation should go beyond the point that the task “mimics real-life environments”, using the presented data on gait analysis as the main point.*

Response: We have included in the discussion, a more in-depth comparison between the evaluation tasks (page 20, line 452-460). The TBC also allows for calculation of additional metrics such as stride time, swing time, asymmetry and arrhythmicity during both non-freezing and freezing behavior in a real-life environment. In addition, the TBC allows measurement of gait transitions into and out of freezing.

*(2) Lines 227-8****:*** *“students t-test” should be “Student t-test.”*

Response: This has been addressed in the revised manuscript (page 11, line 234).

*(3) Lines 229-30: “Paired t-tests were used to compare UPDRS III 230 scores between visits for subjects with repeated visits.” Due to the nature of these data, a corresponding non-parametric test should be used.*

Response: We used a non-parametric test, a paired samples Wilcoxon test and the difference was not significant (*p* = 0.09). We have adjusted the manuscript to address this change (page 11, line 236-7) and (page 12, lines 262-3).

*(4) Lines 507-11: The authors stated that the normalization procedure allowed comparison among individuals with different natural walking speeds. In the sequence, the authors propose that in future studies absolute measures could be tested. From my perspective, (a) speed variability is a feature of this group, and (b) speed normalization is an appropriate procedure for analysis, and so any of these points could not be considered as limitations. The suggestion of using absolute speed is contradictory to the reasons leading to data normalization.*

Response: We agree and appreciate this being brought to our attention, lines (507-11) have been removed to alleviate this contradiction.

**Reviewer #2:**

*The goals of this study were to (1) validate a standardized gait task, the turning and barrier course (TBC), which mimics real-life environments and elicits FOG, (2) discover relevant gait parameters for detecting FOG in Parkinson’s disease in the TBC, and (3) evaluate the effects of 60 Hz and 140 Hz subthalamic deep brain stimulation (DBS) on quantitative measures of non freezing gait and FOG. The paper is interesting, but it needs to have some questions of data analysis answered to support the conclusions.*

*(1) Insert a reference for the method of identifying the phases of the gait from the angular velocity of the shank. Is this method validated to identify gait cycles in patients with Parkinson's disease, especially with freezing episodes? The calculation of the time of the phases is important because it will influence the logistic regression model.*

Response: We agree that the calculation of the time of the gait phases is important. We used a validated method to identify gait cycles (Salarian et al., 2004), and we have clarified and inserted that reference in the manuscript (page 7-8, line 156-61). We have used this method in two other publications (Syrkin-Nikolau et al., 2017, Anidi et al., 2018).

*(2) How did the freezing episodes affect the identification of the gait cycle? Figure 4B shows the difficulty of identifying gait cycles during freezing episodes using the shank's angular velocity
.*

Response: Thank you; we agree and we did not use the standard heel strike to identify the start of a gait cycle for this reason. We used the peak of the angular velocity trace because this was a readily identifiable peak, both with a computer algorithm and visually. Angular velocity peaks were marked as steps only if they exceeded a minimum threshold of 10 deg/s. Therefore, during complete cessation of gait, steps were not identified. It was precisely for this reason, that we identified freezing episodes as a lack of leg swinging, rather than from heel strikes. We have clarified this in the manuscript (pg. 8 line 159-61).

*(3) Detail the reasons for changing the way of calculating the Freeze index.*

Response: We adapted the freeze index window to be more consistent with our stride by stride metrics which were on average 1-2 seconds rather than 4 seconds. Please see page 8, line 170-72.

*(4)* *Why was it analyzed about the rectilinear movement in the Forward Walking task? There is a difference between the tasks because the others have curves.*

Response: The forward walking task was used as a control task that does not typically elicit freezing. In that task we only analyzed rectilinear walking as it reflects the way that patients are usually assessed in clinics. We wanted to compare how well forward walking elicited freezing behavior compared to the turning and barrier course because it is known that freezing is not often elicited during the clinical assessment of gait. This is a large reason why we developed the TBC because of difficulty of eliciting FOG in forward walking.

*(5) A figure would be needed showing the correlations between the variables and the FOG-Q3.*

*Figure 3B could present the values of peak angular velocity shank that was used for the statistic*

Response: We thank the reviewer for this insight and we found that gait arrhythmicity and shank angular velocity did correlate with the FOGQ-3 in addition to the percent time freezing. We have added a figure, Figure 3, and have updated the manuscript (page 12-13, line 269-86).

*(6) Please check the correlation between Arrhythmicity and FOG (Figure 5ª). I don't think there is a correlation given the dispersion of individual data.*

Response: The reviewer is correct, there was no significant correlation between percent time freezing and forward walking (Figure 5a – now Figure 6a). We have made this clearer in the manuscript and in the figure (page 17, line 378-9).

*(7) The analysis of repeated visits is confusing. What are these subjects?*

Response: We had subjects with repeated visits that were, on average, over year apart, 430 ± 112 days (mean ± SD), so we included them as separate visits.

*(8) PLOS authors have the option to publish the peer review history of their article (*[*what does this mean?*](https://journals.plos.org/plosone/s/editorial-and-peer-review-process#loc-peer-review-history)*). If published, this will include your full peer review and any attached files.*

Response: Yes

**References**

Anidi C, O’Day JJ, Anderson RW, Afzal MF, Syrkin-Nikolau J, Velisar A, et al. Neuromodulation targets pathological not physiological beta bursts during gait in Parkinson’s disease. Neurobiol Dis. 2018;120.

Salarian A, Russmann H, Vingerhoets FJG, Dehollain C, Blanc Y, Burkhard PR, et al. Gait assessment in Parkinson’s disease: Toward an ambulatory system for long-term monitoring. IEEE Trans Biomed Eng. 2004;51(8):1434–43.

Syrkin-Nikolau J, Koop MM, Prieto T, Anidi C, Afzal MF, Velisar A, et al. Subthalamic neural entropy is a feature of freezing of gait in freely moving people with Parkinson’s disease. Neurobiol Dis [Internet]. 2017;108(June):288–97. Available from: http://dx.doi.org/10.1016/j.nbd.2017.09.002
